# Cholinergic modulation of the medial prefrontal cortex: the role of nicotinic receptors in attention and regulation of neuronal activity

**DOI:** 10.3389/fncir.2014.00017

**Published:** 2014-03-11

**Authors:** Bernard Bloem, Rogier B. Poorthuis, Huibert D. Mansvelder

**Affiliations:** ^1^Department of Integrative Neurophysiology, Center for Neurogenomics and Cognitive Research, Neuroscience Campus Amsterdam, Vrije UniversiteitAmsterdam, Netherlands; ^2^McGovern Institute for Brain Research, Massachusetts Institute of TechnologyCambridge, MA, USA; ^3^Max Planck Institute for Brain Research, Frankfurt am MainGermany

**Keywords:** acetylcholine, nicotinic receptors, medial prefrontal cortex, attention, neurophysiology

## Abstract

Acetylcholine (ACh) release in the medial prefrontal cortex (mPFC) is crucial for normal cognitive performance. Despite the fact that many have studied how ACh affects neuronal processing in the mPFC and thereby influences attention behavior, there is still a lot unknown about how this occurs. Here we will review the evidence that cholinergic modulation of the mPFC plays a role in attention and we will summarize the current knowledge about the role between ACh receptors (AChRs) and behavior and how ACh receptor activation changes processing in the cortical microcircuitry. Recent evidence implicates fast phasic release of ACh in cue detection and attention. This review will focus mainly on the fast ionotropic nicotinic receptors and less on the metabotropic muscarinic receptors. Finally, we will review limitations of the existing studies and address how innovative technologies might push the field forward in order to gain understanding into the relation between ACh, neuronal activity and behavior.

## Introduction

The prefrontal cortex (PFC) is thought to be important for the highest cognitive processes, including executive functioning (Alvarez and Emory, 2006; Euston et al., 2012), working memory (Funahashi, 2013), decision making (Euston et al., 2012), retrieval from long term memory (Rugg et al., 1996; Tomita et al., 1999), social behavior (Forbes and Grafman, 2010; Avale et al., 2011), emotion (Davidson and Irwin, 1999; Wallis, 2007), personality (Damasio et al., 1994; Kennis et al., 2013) and attention (Miller and Cohen, 2001; Euston et al., 2012). It is thought that subregions mediate different functions. In rodents, the medial part of the PFC (mPFC), has been shown to be important for goal-directed action (Killcross and Coutureau, 2003), working memory (Rossi et al., 2012) and attention (Muir et al., 1996; Passetti et al., 2002; Totah et al., 2009; Euston et al., 2012). This part of the PFC roughly corresponds to the dorsolateral PFC in humans and other primates (Uylings et al., 2003; Vertes, 2004, 2006; Farovik et al., 2008). Lesions of this region result in severe attentional deficits (Muir et al., 1996; Passetti et al., 2003; Kahn et al., 2012) and neuroimaging and electrophysiological studies have shown that this part of the brain is involved in behavioral tasks requiring sustained attention (Gill et al., 2000; Totah et al., 2009; Bentley et al., 2011). Moreover, increasing attentional load by reducing stimulus saliency or introducing distracters increases neuronal activity in the mPFC (Gill et al., 2000).

The PFC receives a dense cholinergic innervation and it is thought that this neurotransmitter plays an important role in the PFC, especially in behavior requiring attention. Acetylcholine (ACh) is a neurotransmitter that is produced in a small number of cells, but has widespread effects throughout the brain (Woolf and Butcher, 2011). Most important for ACh release in the cortex is the basal forebrain, a brain area composed of several cholinergic nuclei, including the nucleus basalis, the septum, the substantia innominata and the diagonal band of Broca (Mesulam, 1995; Zaborszky et al., 1999; Woolf and Butcher, 2011). In addition, ACh is produced in some midbrain nuclei, that is the pedunculopontine nucleus and laterodorsal tegmental area (Mesulam et al., 1983), and in sparsely distributed cholinergic interneurons (Eckenstein and Baughman, 1984; von Engelhardt et al., 2007). In contrast to its local production, the effects ACh exerts on the brain networks are strong and widely distributed. Almost all regions of the brain are innervated by cholinergic neurons and many neurons and glial cells express ACh receptors (AChRs; Van der Zee and Luiten, 1999; Van der Zee and Keijser, 2011; Picciotto et al., 2012). However, it is currently not known how specific the projections of the neurons in the basal forebrain are (Fournier et al., 2004; Chandler and Waterhouse, 2012; Chandler et al., 2013).

To study the effects of ACh on behavior and cognition, researchers have used techniques to measure ACh levels, such as microdialysis and amperometry, and methods to manipulate the cholinergic system, using pharmacology, specific cholinergic lesions and optogenetic manipulations of ACh release. Together, these results indicate that ACh is crucial for attention (Bentley et al., 2011; Klinkenberg et al., 2011), arousal (Metherate et al., 1992; Détári et al., 1999; Platt and Riedel, 2011), learning and memory (Kilgard and Merzenich, 1998; Hasselmo, 2006; Gu et al., 2012) and the sleep-wake cycle (Deurveilher and Semba, 2011; Lin et al., 2011; Platt and Riedel, 2011). It is thought that the effect of ACh depends on its target areas (Everitt and Robbins, 1997; Bentley et al., 2011). In relation to the mPFC, ACh seems mostly involved in attention. Therefore, findings relevant to the role of ACh on attention will be discussed here.

Many studies have demonstrated that pharmacological interventions targeting the cholinergic system or lesions of the basal forebrain affect attention (Jones and Higgins, 1995; Mirza and Stolerman, 2000; Risbrough et al., 2002; Robbins, 2002; Pattij et al., 2007), in addition to other cognitive functions. However, due to the lack of specificity of these methods, it is hard to draw firm conclusions about these, since many processes and brain structures are manipulated simultaneously. Fortunately, more recently it became possible to manipulate the cholinergic system more finely. Studies using local cholinergic lesions or drug administrations and local cholinergic measurements have provided a clearer picture about the role of ACh in the mPFC.

In this review, we will evaluate the evidence that ACh release in the mPFC is involved in attention. The role of AChR, and in particular nicotinic acetylcholine receptors (nAChR), in attention is reviewed and the way in which receptor activation modulates local neuronal activity. In addition, we will address the modulation of these processes by nicotine and smoking and the role of the cholinergic modulation of the mPFC in neuropsychiatric disorders. Finally, an outlook is provided concerning the new possibilities to study the role of ACh release in the mPFC, its relation to behavior and the mechanisms through which this occurs.

## Acetylcholine in the medial prefrontal cortex (mPFC) 

Several lines of evidence indicate that the cholinergic innervation of the mPFC is specifically involved in attention. First, local cholinergic lesions, using the specific immunotoxin 192 immunoglobulin G (IgG)-saporin, result in severely compromised performance in sustained attention tasks (Gill et al., 2000; Chudasama et al., 2004; Dalley et al., 2004). In addition, attention related increases in neuronal activity in the mPFC were absent after cholinergic lesions (Gill et al., 2000).

Secondly, microdialysis studies indicate that attentional tasks are accompanied by increases in ACh concentrations in the mPFC (Passetti et al., 2000; Dalley et al., 2001) that are correlated to the current attentional demands (Kozak et al., 2006). Moreover, recent technological advances (Parikh et al., 2004) made it possible to measure ACh release on a finer timescale. This has revolutionized our understanding of the cholinergic modulation of cortical processes. In particular, the group of Martin Sarter (Parikh et al., 2007; Howe et al., 2013) demonstrated that, whereas cholinergic signaling was traditionally considered to be slow and tonic, there are actually fast transients of ACh in the mPFC during attention tasks. During cues that were detected, rapid elevations in ACh concentrations were observed in the mPFC, whereas in motor cortex, these “transients” were absent. These findings have demonstrated ACh release in relation to a specific cognitive operation and demonstrated that this attentional process involves ACh in the mPFC.

Furthermore, whereas most pharmacological studies concerning the role of AChRs affect many cognitive operations at the same time and cannot differentiate the effects on different brain regions, local infusion of pharmacological agents in the mPFC (Hahn et al., 2003b; Chudasama et al., 2004) can demonstrate an involvement of specific receptors in that region in a certain task. With this method, several groups have demonstrated important roles of the nicotinic (nAChR; Hahn et al., 2003b) and muscarinic (mAChR; Robbins, 2002; Chudasama et al., 2004) receptors in the mPFC in attentional processes.

Finally, it should be noted that the relationship between the mPFC and the basal forebrain is reciprocal. Whereas other cortical areas are also innervated by the basal forebrain, the mPFC is the major source of cortical projections to the basal forebrain (Zaborszky et al., 1997). Hence, it seems that the mPFC is located in a special position with regard to the basal forebrain and that the mPFC-basal forebrain system is critical in mediating sustained attention.

Given the important role of the cholinergic modulation of the mPFC in healthy individuals and the crucial involvement in many neuropsychiatric disorders, it is of great importance to understand the mechanisms by which ACh contributes to cognition and how it influences processing in the microcircuit underlying cognition. Despite the fact that we know that the mPFC and ACh play crucial roles in the ability to focus our attention, very little is known about the exact mechanisms. In particular, the recently discovered phasic cholinergic modulation is very poorly understood. There have been many studies on tonic effects of ACh, suggesting that ACh acts as a neuromodulator and affects attention by increasing the excitability of networks (Picciotto et al., 2012). However, the recent findings that ACh is not only involved in attention by a tonic neuromodulatory role, but also in the mediation of specific cognitive events in single trials—namely cue detection—has posed the question of how short phasic ACh release affects processing in the mPFC network. Recent studies have shed light on how short applications of ACh affect processing in cortical networks and on the role these receptors play in attention. Because the timescale of nAChRs match well with the timescale of the observed phasic release of ACh, most of this review will be devoted to the role of nAChRs in the modulation of processing and the enhancement of attention.

## Cholinergic innervation of the medial prefrontal cortex (mPFC) 

In order to understand the effects of ACh on cortical processing, it is crucial to first know the patterns of innervation. When antibodies for the ACh generating enzyme, choline acetyltransferase (ChAT), became available in the 1980’s, it quickly became clear that the entire cortical mantle is innervated densely with cholinergic axons (Kimura et al., 1980; Bigl et al., 1982; Mesulam et al., 1983; Woolf et al., 1983; Eckenstein and Baughman, 1984; Eckenstein et al., 1988; Wenk, 1997). It was demonstrated that most cholinergic axons originate from the basal forebrain, although cholinergic neurons are also present in the cortex itself (Eckenstein and Baughman, 1984; von Engelhardt et al., 2007). In addition, the PFC receives some fibers from the pedunculopontine nucleus and the laterodorsal tegmental area (Mesulam et al., 1983; Eckenstein et al., 1988), although the functional significance of this is unknown. Although the entire cortex is innervated by ACh, there are laminar differences. In general, layer I–III and layer V are most strongly innervated and layer IV the least. This is due to a layer specificity in the projections of the basal forebrain (Eckenstein et al., 1988) There are differences in this pattern between cortical areas, however, and in the PFC a clear laminar pattern is absent (Eckenstein et al., 1988).

In addition to the pattern of innervation, it is also crucially important to determine what the mode of transmission is. Recently it has been shown that there is both tonic and phasic cholinergic signaling in the mPFC (Parikh et al., 2007). Moreover, it has been long debated whether ACh functions through volume or synaptic transmission (Smiley et al., 1997; Sarter et al., 2009). Both aspects of transmission are crucial for determining the effects of ACh on the mPFC. Recent evidence indicates that most likely both are present (Parikh et al., 2007; Bennett et al., 2012) and that there is a complex interplay of tonic and phasic release, and volume and synaptic transmission, making the precise release parameters crucial for determining the effects on the mPFC.

## Acetylcholine receptors

There are two types of AChRs: the nAChR and mAChR. Both receptors allow ACh to change the electrical activity of the target cells and to affect other processes through intracellular signaling cascades (Dajas-Bailador and Wonnacott, 2004; Gulledge and Stuart, 2005; Intskirveli and Metherate, 2012; Thiele, 2013; Yakel, 2013). However, these receptors function in fundamentally different ways. The nAChR is a pentameric ionotropic receptor, belonging to the cystine-loop superfamily of receptors (Gotti and Clementi, 2004; Changeux, 2012). When ACh binds nAChRs, the channel opens and a direct cationic inward current occurs, which depolarizes the membrane. In contrast, the mAChR is a G-protein coupled receptor and functions through an intracellular signaling cascade (Bubser et al., 2012).

### Muscarinic acetylcholine receptors

There are five different types of mAChRs (M1–M5), all of which are G-protein coupled receptors (Bubser et al., 2012). They can be divided into two principal types, based on the intracellular α subunit type of the G-protein they are bound to. The first main group is made up of the M1, M3 and M5 receptors which interact with Gq/11 proteins, whereas the second group includes M2 and M4 and interacts with Gi/o proteins (Brown, 2010).

In the cortex, mainly M1, M2 and M4 are present (Levey et al., 1991), although M4 has a considerable lower expression than the first two. Through a variety of intracellular signaling cascades, mAChR activation affects the functioning of many ion channels, resulting in changed conductances of mainly potassium and calcium channels (Thiele, 2013). In general, M1 activation results in a lower potassium conductance, whereas M2 and M4 result in an increase of potassium conductance and a decrease of calcium conductance. Gulledge et al. (Gulledge and Stuart, 2005; Gulledge et al., 2007, 2009) have demonstrated that cortical layer V pyramidal neurons are strongly modulated by M1 receptors in a complex fashion. Phasic ACh application hyperpolarized and/or depolarized these neurons, whereas tonic presence of ACh had the opposite effect. Importantly, the intracellular signaling pathway mediated effects of mAChR binding have a slow timescale compared to the effects mediated by nAChR, which result in a direct inward current with a fast onset and a slower duration (Gulledge et al., 2007).****

### Nicotinic acetylcholine receptors

nAChRs are ligand-gated ion channels with a pentameric structure and are composed of five subunits. There are 12 neuronal subunits (α2–α10 and β2–β4) (Gotti and Clementi, 2004) and, consequently, there are many types of receptors that can be formed (Gotti et al., 2006). There are two main subfamilies of nAChRs. The first is the homopentameric receptors that are formed by 5 α subunits. Both ACh and nicotine, an exogenous ligand of the nAChR, bind to the interfaces of the opposite sides of the α subunits. Second, there are heteropentameric receptors that are composed of two α subunits, carrying the principle ligand binding site, and two β subunits, containing the complementary binding (Gotti et al., 2006). In addition, there is a fifth subunit that does not contribute to ligand binding but which can nevertheless influence the characteristics of the receptor. In the cerebral cortex, there are only two main types of receptors present (Alkondon and Albuquerque, 2004). First, there are homopentameric receptors composed of five α7 subunits. Secondly, there are heteromeric receptors that contain 2 α4 subunits, 2 β2 subunits and a fifth subunit, which can be α4, β2 or α5 (Albuquerque et al., 2009). There are important differences between the different nAChRs and this also holds true for the two types present in the cerebral cortex.

All nAChRs are cationic selective channels, permitting a flow of Na^+^, K^+^ and Ca^2+^, thereby depolarizing the membrane. However, there are substantial differences in the conductances for these individual ions in the different receptor types (Fucile, 2004). It has been shown that especially the homopentameric α7 nAChR is permeable to calcium and that the addition of the α5 subunit to the heteropentameric α4β2 nAChR greatly increases its calcium conductance (Fucile, 2004). Calcium conductance is an interesting property of nAChR because this links nAChR activation to intracellular signaling pathways (Dajas-Bailador and Wonnacott, 2004; Gubbins et al., 2010) and because it mediates the effect of presynaptic nAChR stimulation on increased neurotransmitter release (Sharma and Vijayaraghavan, 2003; Dickinson et al., 2008). Despite the fact that the α4β2 nAChR has a substantially lower calcium conductance, it should be noted that also activation of this receptor can induce intracellular calcium signaling through its association with voltage operated calcium channels (VOCCs; Dajas-Bailador and Wonnacott, 2004). Another important difference between the two main groups of nAChRs is their affinity to ACh (Clarke et al., 1985). In contrast to the heteropentameric receptors, that have a nanomolar affinity to ACh, homopentameric receptors have an affinity in the micromolar range (Gotti et al., 2006). This is one of the reasons why it has been suggested that homopentameric α7 receptors are located in synapses and that α4β2* nAChRs (* denotes the presence of a fifth accessory subunit) are located extrasynaptically and are activated by volume transmission (Bennett et al., 2012).

An interesting property related to the differences in affinity is the desensitization of both types of receptors. Whereas the α7 nAChR desensitizes fast to high concentrations of ACh (McGehee and Role, 1995), a radically different picture emerges when looking at low agonist concentration desensitization. At agonist concentrations that are insufficient for receptor activation, desensitization can be observed in high-affinity α4β2* nAChRs receptors. This process has been termed “high-affinity desensitization”, to distinguish it from “classical desensitization” (Giniatullin et al., 2005). In other words, the α7 nAChR desensitizes quickly to high agonist concentrations, and the α4β2* nAChRs desensitizes much slower but also in response to much lower ACh concentrations (Mansvelder et al., 2002). Desensitization is an important property of nAChRs because it has been shown that realistic concentrations of nicotine, after the smoking of only one cigarette (Henningfield et al., 1996; Matta et al., 2007; Rose et al., 2010), desensitize high-affinity nAChRs in the ventral tegmental area (VTA) and thereby contribute to the addictive properties of nicotine (Mansvelder et al., 2002; Wooltorton et al., 2003).

There are also important differences in the timescale of the currents that are flowing through the channels and the pharmacological profile of the receptors. Hence, the two main types of nAChRs can be distinguished easily based on their sensitivities to particular pharmacological agents and the timescale of their activation (McGehee and Role, 1995).

Finally, the accessory α5 subunit has an important influence on the heteropentameric receptor. In addition to the already mentioned increase in Ca^2+^ conductance, this subunit has also been shown to increase conductance and the sensitivity to nicotine (Ramirez-Latorre et al., 1996), to prolong inward currents in response to persistent nicotine application (Bailey et al., 2012) and potentially to influence the receptor localization (Gotti and Clementi, 2004). Furthermore, recently it was also demonstrated that the α5 subunit influences the expression of the α4 subunit in the VTA (Chatterjee et al., 2013).

## Role of nicotine receptors in behavior

During attention tasks there is a release of ACh in the mPFC which is associated both with attentional effort and with cue detection (Passetti et al., 2000; Parikh et al., 2007). Recently, mice lacking specific nicotinic subunits were tested in the 5-choice serial reaction time task (5-CSRTT; Robbins, 2002), an attentional task for rodents in which the animals have to respond to 5 different cue lights by making a nosepoke in the corresponding hole in order to obtain food rewards. The results indicate that β2 subunits in the prelimbic cortex are necessary for cue detection, as mice lacking these subunits make more errors of omission in this task and reexpression of these subunits in the prelimbic cortex was sufficient to rescue behavior (Guillem et al., 2011). This is the first time that attention problems have been demonstrated in these mice. Although the authors did not find altered behavior in mice lacking the α7 subunit, others have reported that α7 knock-outs do have attentional deficits as apparent by an increase in omissions and a decrease in accuracy (Young et al., 2004, 2007; Hoyle et al., 2006). A possible explanation for this discrepancy is that in these latter experiments the mice performed more trials. Hence, it could be that the demands on sustained attention were higher thereby revealing the phenotype. Moreover, in the experiments of Guillem (Guillem et al., 2011) the mice made relatively more omissions, making it possible that the differences were masked by a ceiling effect. Nevertheless, the fact that they did find an effect on omissions in the β2 knock-out mice suggests that they were able to measure differences in attention behavior between different phenotypes and that probably the phenotype of α7 knock-outs is more subtle.

Although the role of the β2* nAChRs in attention behavior has not been tested before with the use of mice lacking these subunits, there have been attempts to study them using a pharmacological approach. In other studies using the same behavioral task, it was found that pharmacological blockade of β2* nAChRs did not affect task performance in rats (Grottick and Higgins, 2000; Hahn et al., 2011) and in mice (Pattij et al., 2007). Therefore it was concluded that these receptors are not involved in cue detection. There are several possible explanations for the discrepancy between these findings. First, there could be species differences explaining the lack of effect in rats. Secondly, differences could be due to the concentration of antagonist applied and residual effects of ACh through nAChRs. It is not completely known how high the antagonist concentration is in the mPFC when it is administered systemically. In addition, in electrophysiological recordings there is not a full blockade of the inward currents (Guillem et al., 2011; Poorthuis et al., 2013a) after local ACh application in the presence of the β2* nAChRs antagonist, dihydro-β-erythroidine (DHβE), that was used in the rat studies. In addition, knocking out genes can induce compensatory effects and developmental changes. Indeed, it is known that mice lacking β2 subunits have an upregulation of muscarinic excitability (Tian et al., 2011).

Interestingly, it has also been demonstrated that the α5 subunit, which is present on layer VI pyramidal neurons, is necessary for normal attention behavior (Bailey et al., 2010). In contrast to β2 knock-out mice, mice lacking the α5 subunit have a reduced accuracy in the 5-CSRTT and only a small, but not significant, effect on omissions. Since α5 and β2 subunits form nAChRs only on layer VI pyramidal cells, it could be that the effect on omissions is dependent on nAChRs that do not have the α5 subunit. In contrast, the effect on accuracy in α5 knock-out mice could be due to differences that are due to the role of the α5 subunit in development, as mice lacking this subunit have neurons with shorter apical dendrites (Bailey et al., 2012). Alternatively, it could be that β2* nAChR are specifically involved in the mediation of the effects of cholinergic transients, whereas α5β2* are more important for tonic effects of ACh. This could well be the case, since that would mean that the timescale of their activation would match the release mode.

In addition to the knock-out approach to probe the involvement of specific receptors in this task, other studies have also used pharmacological methods. Most of these have used systemic administration of nicotinic and/or muscarinic drugs and are hard to interpret since nAChRs throughout the brain are activated. However, a small number of studies have infused cholinergic drugs locally into the mPFC, thereby generating important data regarding the cholinergic modulation of this brain area. In one study, nicotine was infused systemically or locally into the mPFC or hippocampus and attention behavior in the 5-CSRTT was compared between these conditions (Hahn et al., 2003b). This study elegantly showed that the effects of systemic nicotine on the accuracy in the task could also be observed after local infusion of nicotine. In contrast to what one would expect on the basis of studies using knock-out mice (Guillem et al., 2011), they did not find that nicotine in the mPFC could replicate the effects of systemic nicotine on omissions. There was no effect of nicotine on the dorsal hippocampus. The same authors also performed another study in which they investigated the contribution of heteromeric and homomeric nAChRs to the effects of nicotine on the 5-CSRTT using the specific antagonists DHβE and methyllycaconitine (MLA; Hahn et al., 2011). Based on co-application of these antagonists and nicotine, they concluded that the effects of nicotine are mediated by α7 nAChRs and not by β2* nAChR. A more recent study, in which nicotinic agonists were used, shows however that the attention enhancing effects of nicotine are also seen with specific β2* nAChR agonists, but not with α7 nAChRs agonists (Young et al., 2013).

To summarize, although there is plenty of evidence showing that prefrontal ACh is crucial for attention behavior and that nAChRs are involved in performance during the 5-CRSTT, it is currently not completely clear what the role of different types of receptors are and how exactly they change the number of omissions and accuracy. Interpreting the results is complicated by the fact that there are many small differences in task design and because of problems with interpreting systemic administration and knockout studies. Nevertheless, recent results are clearly showing an involvement of the β2* nAChRs in cue detection during the 5-CSRTT (Guillem et al., 2011).

## Cholinergic modulation of cortical circuitry

The cortex is a six-layered structure (I–VI) (Douglas and Martin, 2004), although the rodent PFC misses the classical input layer IV (Uylings et al., 2003). In addition, there is a second organizational principle, called cortical columns (Mountcastle, 1997; Markram et al., 2004) in which neurons often have similar receptive field properties. Although the existence of cortical columns in all regions of the cortex is controversial (Horton and Adams, 2005), it is a useful concept to understand processing in the cortical circuitry. Within these different layers, there are excitatory, glutamatergic pyramidal neurons and inhibitory, GABAergic interneurons. These are thought to modulate processing locally by inhibiting the activity of the pyramidal neurons, thereby shaping processing in the local microcircuitry (Markram et al., 2004; Huang et al., 2007; Isaacson and Scanziani, 2011). Both of these groups of neurons can be further divided into many subclasses on the basis of morphology, electrophysiological firing pattern, projection targets and molecular characteristics (Ascoli et al., 2008; DeFelipe et al., 2013).

Although it is not known how exactly information is processed in cortical circuits, many studies have looked into the connectivity and information flow in the cortical circuitry of primary sensory areas (Armstrong-James et al., 1992; Thomson et al., 2002; Hirsch and Martinez, 2006; Feldmeyer, 2012; Constantinople and Bruno, 2013). It remains to be seen whether these findings can be generalized to higher order cortical areas such as the PFC. Based on this work, a general model of information flow within cortical circuits has been proposed. To describe processing, it is useful to describe the direction of information flow in the cortical hierarchy. Conceptually this is easiest to understand in the visual cortex (Hubel and Wiesel, 1977). In this system, there is a clear hierarchy of cortical areas that process visual information in which the receptive field properties get bigger and more complex throughout the visual system (Hubel and Wiesel, 1962, 1965; Moran and Desimone, 1985; Felleman and Van Essen, 1991). There are three different possible “directions” in which processing can occur (Lamme et al., 1998). First, there is feedforward processing, meaning that sensory information entering the cortex is processed according to these hierarchical steps in a bottom up fashion. In contrast, there is feedback processing (Lamme et al., 1998; Lamme and Roelfsema, 2000), referring to a modulation of the processing of incoming information by hierarchically higher brain areas. Examples are top-down attention, predictions and expectations (Lamme and Roelfsema, 2000). Finally, there is lateral processing (Lamme et al., 1998) referring to horizontal integration or competition at a given level of the hierarchy (Gilbert and Wiesel, 1989; Adesnik and Scanziani, 2010).

In sensory cortical areas, feed-forward information enters the cortex from the thalamus and targets layer IV (Castro-Alamancos and Connors, 1997; Douglas and Martin, 2004). Layer IV excitatory neurons project to the superficial layer II and III, which subsequently send information to the deep layer V (Gilbert and Wiesel, 1979; Thomson et al., 2002; Thomson and Bannister, 2003). Layer V innervates layer VI and sends a signal back to the superficial layers. Also, this layer and layer VI project strongly to subcortical structures such as the thalamus and the basal ganglia (Gabbott et al., 2005; Olsen et al., 2012). For this reason, they are sometimes referred to as the cortical output layers In contrast, layer II and III project mainly to other cortical areas (Adesnik and Scanziani, 2010; Little and Carter, 2012). Finally, layer I is very different from the other layers, since the density of neurons is extremely low (Meyer et al., 2010) and all neurons are GABAergic interneurons (Jiang et al., 2013). It is thought that thalamic feedback signals are send to layer I and that this modulates processing in the cortical column (Rubio-Garrido et al., 2009; Letzkus et al., 2011; Cruikshank et al., 2012).

As stated before, this model is based on information from sensory cortical areas and it remains to be determined whether it holds for the mouse mPFC. Furthermore, it is a simplified model since, for example, also in the barrel cortex layers V and VI receive monosynaptic inputs from the thalamus (Agmon and Connors, 1991; Constantinople and Bruno, 2013). One important difference between the PFC and the sensory cortices is that the rodent PFC does not have a layer IV. Instead, inputs from higher order thalamic relay nuclei (Sherman, 2012) target layer II/III and V. In addition, the superficial layers are modulated, like other cortical areas, by nonspecific thalamic projections (Little and Carter, 2012). Another feature of the PFC which distinguishes it from other cortical areas is the strong recurrent connectivity (Wang et al., 2006) and persistent firing outlasting stimulus presentations (Zhang and Séguéla, 2010; Yang et al., 2013) that can be observed in this area. Hence, we are only beginning to understand how information flows in the cortical microcircuitry. Nevertheless, a picture is emerging how ACh modulates the flow of information in the cortex.

On a network level, basal forebrain stimulation in anesthetized animals results in a desynchronized state of field potentials (Goard and Dan, 2009; Kalmbach et al., 2012) and neuronal firing in the basal forebrain is correlated with a reduction in low frequency and an increase of high frequency oscillations in the cortex (Duque et al., 2000; Manns et al., 2000). Since these frequency bands are related to the state of arousal and cortical activation (Uhlhaas et al., 2008; Deco and Thiele, 2009; Wang, 2010; Cachope et al., 2012), ACh has long been considered a neuromodulator that is involved in setting the state of arousal. Mechanistically, it was shown that ACh activated cortical mAChRs on pyramidal neurons (Gulledge et al., 2009), thereby shifting firing modes from bursting to tonic and changing low frequency high amplitude oscillatory activity to high frequency low amplitude on a network level (Metherate et al., 1992).

Other studies have looked at the effect of ACh on the direction of the flow of information in the cortex. Again, these studies have been performed in sensory areas because in these regions, neuronal responses could be related to sensory stimulation. One of the dominant effects that has repeatedly been demonstrated is the enhancement of feedforward thalamic input into the sensory cortical areas. In layer IV, ACh increases the gain and reliability of neuronal responses in layer IV of the visual cortex (Goard and Dan, 2009; Soma et al., 2012, 2013), an effect which is mediated by heteromeric nAChRs (Roberts et al., 2005; Disney et al., 2007). In the barrel cortex, a similar effect was observed (Oldford and Castro-Alamancos, 2003). In layer II and III, the picture is more complex. In general, cholinergic modulation reduces firing rate in these layers by increasing GABAergic inhibition through mAChRs and nAChRs (Disney et al., 2012; Alitto and Dan, 2013; Soma et al., 2013), although reliability of encoding and modulation by presented stimuli sometimes increased at the same time (Goard and Dan, 2009; Soma et al., 2013). Interestingly, it has recently been reported that the cortical depression associated with whisker trimming is accompanied by an increase of heteromeric receptors on interneurons in layer II/III and that blocking these receptors can prevent the cortical depression. This suggest that heteromeric receptors in layer II/III are required for regulating the responsiveness of the somatosensory cortex (Brown et al., 2012). Intracortical projections, which are thought to connect superficial layers between different cortical columns are also inhibited by ACh through mAChRs (Kimura and Baughman, 1997). Based on this finding and the reduced activity in the superficial layers, it has been suggested that ACh reduces horizontal processing through cortico-cortical interactions (Hasselmo and Giocomo, 2006). Indeed it has been observed in slices, in vivo animal experiments and in humans that the spatial spread of excitation in response to stimuli is reduced in the presence of elevated levels of ACh (Kimura et al., 1999; Silver et al., 2008). This effect could have a sharpening effect on tuning curves of receptive fields and the discriminability of sensory stimuli (Roberts et al., 2005; Thiele et al., 2012). Also, the combination of reduced lateral interactions and an increased sensitivity to thalamic inputs could increase the networks sensitivity to incoming information and increase the signal to noise ratio. This effect is also observed with enhanced attention (Briggs et al., 2013). Therefore, this could be one of the core mechanisms through which ACh modulates selective attention (Hasselmo and Giocomo, 2006; Deco and Thiele, 2011; Hasselmo and Sarter, 2011). The effect of ACh on the deeper layers V and VI is less understood in functional terms. However, also in deep layers both pyramidal and interneurons are modulated by nAChRs and mAChRs (Gulledge et al., 2007; Kassam et al., 2008; Poorthuis et al., 2013a) and both response suppression and facilitation can be observed (Soma et al., 2013). Finally, in layer I, all interneurons contain heteromeric and/or homomeric nAChRs (Christophe et al., 2002; Alitto and Dan, 2013). Since these neurons inhibit both layer I-III interneurons and layer II/III pyramidal cells, the effect of cholinergic layer I activation is complex and can inhibit as well as disinhibit pyramidal cells in deeper layers (Letzkus et al., 2011; Arroyo et al., 2012; Bennett et al., 2012; Cruikshank et al., 2012; Jiang et al., 2013).

## Cholinergic modulation of the medial prefrontal cortex

Despite the fact that the effects of ACh, as described above, are found in sensory cortices, there are reasons to believe that the cholinergic modulation of the mPFC occurs in a similar manner. Autoradiographical measurements of the localization of mAChRs and nAChRs do not show big differences in receptor localization between different cortical regions (Clarke et al., 1984, 1985; Spencer et al., 1986). In addition, there is evidence that some of the principles outlined above also hold true for the mPFC. For instance, also in the mPFC layer V pyramidal neurons are prominently modulated by M1 (Gulledge et al., 2009) whereas layer II–III pyramidal neurons are not. Moreover, also in the mPFC the release of other neuromodulators is strongly increased by nicotinic stimulation (dos Santos Coura and Granon, 2012).

In contrast to other cortical regions, where thalamic axons target mainly layer IV, in the mPFC they target layer III and V (Rotaru et al., 2005), as layer IV is nonexistent. It has been demonstrated that after lesioning of the thalamic nucleus targeting the PFC, the mediodorsal thalamus (MDT), there is a 40% reduction of high affinity binding sites, suggesting a strong heteromeric nAChR presence on the thalamocortical terminals (Gioanni et al., 1999). In addition, this study demonstrated that nicotine induces a strong glutamate release in the PFC and that an iontophoretic nicotine application enhanced the response to MDT stimulation in all layers. Moreover, it was demonstrated that nicotine increases spontaneous release of glutamate from thalamic inputs onto layer V neurons (Lambe et al., 2003). In contrast, in layer II/III mAChR and nAChR seem to have opposing effects on glutamatergic inputs, although the percentage of neurons modulated in this layer is rather low (Vidal and Changeux, 1993). Given these findings and the increase of coding reliability that is observed in sensory areas after nAChR stimulation (Disney et al., 2007; Goard and Dan, 2009; Soma et al., 2012), one could speculate that an enhancement of thalamocortical processing is a dominant effect of nAChR stimulation in the mPFC. Interestingly, heteromeric receptors on these terminals were not reexpressed in (Guillem et al., 2011), demonstrating that it is unlikely that β2*-nAChRs on thalamic inputs play a role in cue detection in this task.

**Figure 1:**
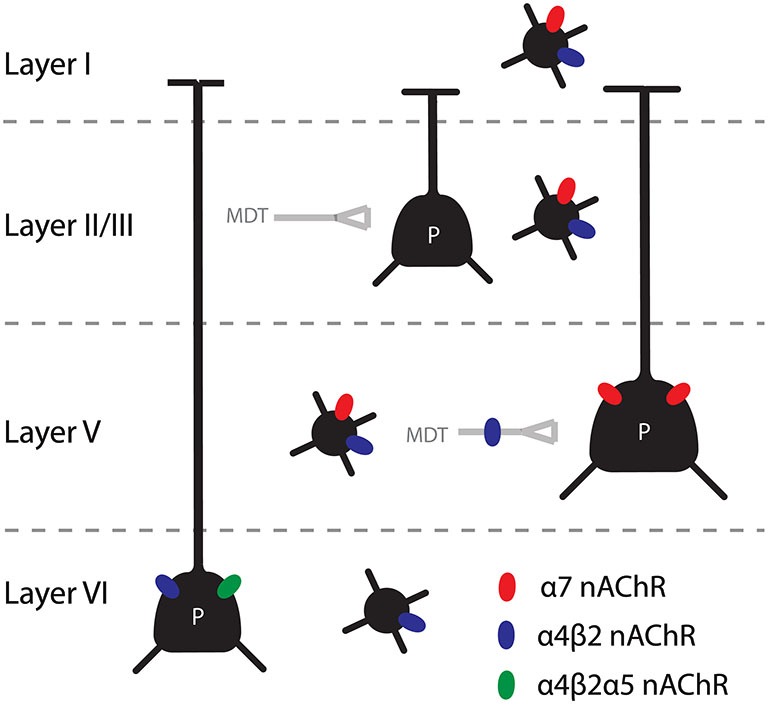
**Summary of the main findings concerning nAChRs in the mPFC.** Pyramidal cells in layer V and VI are directly modulated by nicotinic receptors, through α7 and β2* nAChRs respectively. In addition, the thalamic input to layer V is heavily modulated by β2* nAChRs. Interneurons in all layers contain nAChRs, although the distribution of homomeric and heteromeric receptor is different for different interneuron types and the different layers.

In addition to these presynaptic receptors, β2*-nAChRs were also found postsynaptically on cells in the mPFC (Figure 1). It was found that there is a strong presence of α4β2α5 nAChRs on pyramidal cells in layer VI and α4β2* nAChRs on interneurons in all layers (Poorthuis et al., 2013a; Poorthuis and Mansvelder, 2013). Given the finding that reexpression of β2 subunits in the prelimbic cortex could rescue the phenotype of β2 knockout mice, it is most likely that these receptors are crucial for cue detection in the 5-CSRTT. This would suggest that during a sustained attention task, ACh increases inhibition in the mPFC through nAChRs and increases pyramidal cell activity in layer VI. These pyramidal neurons feed back to the thalamic inputs of the mPFC (Gabbott et al., 2005). In the visual cortex these layer VI pyramidal neurons have been shown to modulate the gain of incoming thalamic information (Olsen et al., 2012). It would be interesting to disentangle the contribution of prelimbic interneurons and layer VI pyramidal cells in an attention task to further narrow down the specific β2* nAChRs that are required for cue detection. Homomeric receptors were also found in pyramidal cells of the mPFC in a layer and neuronal subtype specific manner. Interestingly α7 receptors were reported to be present on layer V pyramidal neurons (Poorthuis et al., 2013a). To our best knowledge, this is the first demonstration of a homomeric nAChR presence on layer V pyramidal cells. During development, there is a transient upregulation of the expression of the α5 subunit in the cortex (Winzer-Serhan and Leslie, 2005). The first months there is a particularly high expression in layer VI, with a peak around 2 weeks after birth. It was shown that this is also the case in the PFC and that these α5 expressing neurons are pyramidal neurons projecting to the MDT (Kassam et al., 2008). In addition, some cells in layers II-V express this accessory subunit. These cells are thought to be interneurons, based on electrophysiological recordings and post-hoc single cell reverse transcription polymerase chain reaction (RT-PCR; Porter et al., 1999).

As in other cortical areas, non fast spiking interneurons are modulated by mAChRs and nAChRs stimulation (Kawaguchi, 1997; Gulledge et al., 2007; Poorthuis et al., 2013a). In contrast, it is unclear how exactly fast spiking interneurons are modulated by ACh. There have been reports that fast spiking interneurons are unresponsive to cholinergic stimulation (Kawaguchi, 1997; Gulledge et al., 2007) but it has also been published that fast spiking interneurons are inhibited through mAChR in layer V of the visual cortex (Xiang et al., 1998), that mAChR activation inhibits GABA release from fast spiking cells on pyramidal cells in the somatosensory cortex (Kruglikov and Rudy, 2008) and that α7 nAChRs are present on fast spiking interneurons in layer I-V. In layer I all neurons have nAChRs, as described above. A consequence of the nicotinic stimulation of interneurons is that nicotine has been shown to increase the inhibition of layer V pyramidal neurons (Couey et al., 2007). Hence, interneurons in all layers, except for layer VI contain a mixed profile of nAChRs. This includes both fast spiking and non-fast spiking interneurons although there are differences in nAChRs in these two populations in the different layers.

Together, these results show that the models of cholinergic modulation from sensory areas are at least useful to understand the cholinergic modulation of the mPFC. Nevertheless, in order to understand the way AChRs mediate the effects of phasic ACh release in the mPFC, it will be crucial to study the receptor localization and their effects on network physiology into more detail.

Given these findings, one could speculate about the functional role of nAChRs in the modulation of mPFC activity by ACh. It seems that nAChR stimulation results in an increase of the inhibitory tone of the mPFC network. In addition, there seems to be a strong increase in the processing of thalamic information. Together this could mean that nAChR stimulation would “reset” the network so that new incoming information can be processed. This would fit well with the model that was proposed by Sarter (Sarter et al., 2005; Howe et al., 2013) in which short increases in ACh would mediate an attentional shift, or more precisely: a shift from perceptual attention to the activation of response rules allowing the expression of a behavioral response. Furthermore, as in sensory cortices the data support the model that ACh reduces the functional connectivity of corticocortical projections. In other words, also in the mPFC there is an increased drive from the thalamus whereas the superficial layers, that mediate most of the corticocortical connectivity, are inhibited. In the deep layers, it was recently found that nAChR activation increases spontaneous activity in acute brain slices. Based on the connectivity of layers V and VI, this would suggest that the activation of nAChRs in the mPFC by ACh increase the drive from this region on subcortical structures. Since layer V strongly connects to the striatum, it could be that the activation of this layer is important in the initiation of the behavioral response after the mPFC has detected the cue. In contrast, layer VI projects back to the MDT, which could modulate the gain of the thalamic inputs. To determine the effects of activation of these layers, it will be necessary to perform *in vivo* experiments in which the activity in different layers will be measured and/or manipulated.

Since it is known that the basal forebrain gets activated in response to salient events (Lin and Nicolelis, 2008) and that there are strong projections to this region from subcortical areas like the nucleus accumbens (St. Peters et al., 2011) and the amygdala (Jolkkonen et al., 2002), it seems that phasic cholinergic signaling in the mPFC is important for signaling salient information. In other words, when important information regarding potential rewards or dangers are presented or expected, ACh might update the internal goals, the direction of attention, the content of working memory and/or a change in behavior.

It remains to be determined how this links to the effects of ACh on sustained attention. It could be that ACh influences sustained attention through this fast signaling mode and that when sustained attention fades, this is reflected by a reduction in the size or frequency of cholinergic transients. Alternatively, the effects of ACh on sustained attention might be independent of fast cholinergic transients and instead related to tonic release of ACh. Finally, there might be a complex interplay between tonic and phasic effects.

## Exogenous nAChR activation: activation and desensitization by nicotine

Although the endogenous ligand for nAChRs is ACh, many people use a drug that contains an exogenous ligand for this receptor, namely nicotine, in the form of smoking of tobacco. Since there is evidence that nicotine influences attentional performance (Mirza and Stolerman, 2000; Hahn et al., 2003a; Levin et al., 2006; Heishman et al., 2010) and that at least a part of these effects are mediated by prefrontal nAChRs in rats (Hahn et al., 2003c), it is interesting to see how realistic concentrations of nicotine affect cholinergic signaling through nAChRs in the mPFC. It was found (Poorthuis et al., 2013b) that nicotine activates nAChRs and thereby influences network activity, although the main effect of nicotine is actually a desensitization of nAChRs. Especially heteromeric nAChRs desensitize strongly in the presence of 300 nM nicotine, a concentration that is found in the brain after the smoking of just one cigarette for over 10 min. For this reason, it was concluded that nicotine interferes strongly with cholinergic signaling through nAChRs. In addition to the activating and desensitizing properties of nicotine when it binds to the nAChRs, it has also been shown that nicotine can induce persistent changes in gene expression in multiple brain areas, including the mPFC (Mychasiuk et al., 2013), and that it strongly influences the presence of high affinity nicotine receptors in the brain (Marks et al., 1992; Buisson and Bertrand, 2001). The mechanisms behind this are still controversial (Vallejo et al., 2005; Govind et al., 2012) but it has been firmly established that this is the case.

At the behavioral level, although the evidence for an effect of nicotine on attention is strong, the precise conditions under which this can be observed are still under debate. Although nicotine seems to improve cognition in certain patient populations including schizophrenia, ADHD and dementias (Newhouse et al., 2004; Potter and Newhouse, 2008; D’Souza and Markou, 2012), the evidence for an attention enhancing effect in healthy populations is scarce (Newhouse et al., 2004; Heishman et al., 2010). Moreover, people that are addicted to smoking function better when they are not in a state of abstinence (Kleykamp et al., 2005; Vossel et al., 2011) although this seems to reduce a cognitive deficit associated with the abstinence rather than to really improve attention. Importantly, in humans it is unlikely that smokers represent an unbiased sample of the population. Rather, attentional problems or other cognitive deficits might already be present (Rigbi et al., 2008). Also, mutations in the genes coding for the nAChR subunits influence smoking behavior itself (Picciotto and Kenny, 2013). To circumvent these problems, animal work provides an outcome. In sustained attention tasks, many groups have shown that acute nicotine administration can improve performance (Grottick and Higgins, 2000; Stolerman et al., 2000; Hahn et al., 2003a; Young et al., 2013) although there are still some discrepancies between the different findings (Mirza and Stolerman, 1998; Robbins, 2002). Importantly, the age and duration of nicotine administration have been found to be important parameters (Counotte et al., 2012b). Rats that received nicotine during adolescence had attentional difficulties in adulthood, an effect that was not observed when nicotine was delivered during adulthood (Counotte et al., 2011, 2012a).

There seem to be big differences between acute and chronic nicotine administration. Especially at an early age, the network is prone to adapt quickly. Because nicotine use in humans often starts during puberty and is occurring during prolonged periods, it is likely that the effects of nicotine on cognition in humans are different from what was observed in slices. For this reason it is hard to explain the cognitive effects of smoking from the data on desensitization. Nevertheless, it suggests that nicotine does not exert its effects as an agonist, but rather as an agent that desensitizes β2* nAChRs. Recently, several groups have started disentangling the activating and desensitizing effects of nicotine in attention. Levin and Rezvani have administered nAChR antagonists and an agonist that mainly desensitizes high affinity nAChRs and found that attention can be improved by these drugs (Levin et al., 2013; Rezvani et al., 2013). Therefore this would suggest that the attention enhancing effects of nicotine are actually mediated by a desensitization of nAChRs. This raises the question, however, why mice lacking β2* nAChRs were shown to have an attentional deficit and the administration of nAChR antagonist mecamylamine increases the number of omissions (Pattij et al., 2007). To conclude, although there is a lot of evidence that nicotine influences attentional performance, it is still under debate what the exact conditions are under which it improves or decreases attention and what the mechanisms are through which it does so.

## The role of cholinergic modulation of the medial prefrontal cortex (mPFC) in neuropsychiatric and neurodegenerative disorders

There are many neuropsychiatric disorders associated with dysfunctions in the cholinergic system and the mPFC. It is beyond the scope of this review to detail all mechanisms of these disorders, but findings relating to the role of the mPFC, ACh and attention will be highlighted shortly.

Given the studies mentioned above, it is no surprise that attention deficit hyperactivity disorder (ADHD) is associated with dysfunctions in the mPFC and the cholinergic system. ADHD is characterized, among others, by a decreased top down control, inattention and impulsive acts, all of which are strongly linked to the mPFC and ACh (Robbins, 2002; Sarter and Paolone, 2011; Ohmura et al., 2012). Furthermore, nicotine itself can increase cognitive performance in ADHD patients (Newhouse et al., 2004; Levin et al., 2006) and since recently, clinical trials are being performed to test the efficacy of nAChR subtype specific agonists to increase cognitive performance in ADHD patients (Bain et al., 2013; Jucaite et al., 2014).

In addition to ADHD, schizophrenia is also associated with disturbances in the cholinergic system and the mPFC (Weinberger and Berman, 1996; Minzenberg et al., 2009; Brooks et al., 2011, 2012). Schizophrenia patients have deficits in PFC dependent cognition, such as working memory (Forbes et al., 2009) and behavioral flexibility (Leeson et al., 2009) and have alterations in the microcircuitry of the PFC, in particular in interneurons (Lewis et al., 2005; Uhlhaas and Singer, 2010). In addition, multiple ACh receptor types have been linked to the disease (Raedler et al., 2003; Wallace and Bertrand, 2013). Although the relation is far from clear, a number of observations have been made that establish a link between schizophrenia and the α7 nAChR. First, it is expressed to a lower degree in schizophrenia patients (Guan et al., 1999; Young and Geyer, 2013). Moreover, in mice this receptor is linked to sensorimotor deficits that are also found in schizophrenia patients and their healthy family members (Martin and Freedman, 2007). Also, the part of the genome coding for this receptor is linked to schizophrenia. Finally, it is known that schizophrenia patients participate in heavy nicotine searching behavior, which could compensate for the lower expression of α7 receptors, and that nicotine, in addition to more selective α7 agonists, can improve cognitive functioning in these patients (Olincy et al., 2006; Wallace and Bertrand, 2013).

Obviously, another psychiatric disorder associated with nAChRs in particular is addiction. Of all drugs, nicotine is used most extensively and it is associated with a significant social and economic burden for society (Dani and Balfour, 2011; De Biasi and Dani, 2011; Picciotto and Kenny, 2013). Fundamentally, addiction is not an attentional disorder. However, addiction is linked to changes in functioning of the mPFC and behavioral control (Van den Oever et al., 2010; Goldstein and Volkow, 2011) and it has been shown that attention is impaired after nicotine exposure (Counotte et al., 2011). Moreover, people using nicotine often report attentional benefits although it’s not clear to what extent this is due to a relief from withdrawal symptoms or acute effects (Heishman et al., 2010).

Finally, given the fact that lesion, electrophysiological and pharmacological studies strongly indicate that ACh is a key neurotransmitter in memory function (Deiana et al., 2011), it is not surprising that another disorder strongly linked to cholinergic functioning is Alzheimer’s disease (AD). Because of reports (Davies and Maloney, 1976) of strong cholinergic cell loss in the septum and basal forebrain of Alzheimer’s patients, early theories of AD emphasized a cholinergic involvement. As later it became clear that cholinergic cell loss does not occur in early stages of the disorder, it became clear that this cannot account for AD as an etiological factor (Pinto et al., 2011; Schliebs and Arendt, 2011). However, widespread cholinergic cell loss is still considered a major aspect of AD (Micheau and Marighetto, 2011). Another important link between AD and cholinergic signaling is through the nAChR (Buckingham et al., 2009; Jürgensen and Ferreira, 2010). It has been found that AD patients have strongly reduced levels of cortical α4β2 nAChRs (Kellar et al., 1987; Sparks et al., 1998; Perry et al., 2000). In addition, it was demonstrated that the major constituent of the extracellular placques, amyloid-beta, can directly interact with nAChRs and interfere with their functioning (Dineley, 2007). Although there are still a lot of questions about these interactions and about cholinergic cells loss in AD, it is clear that cholinergic dysfunction plays an important role in the memory and attention problems in AD patients (Brousseau et al., 2007; Pinto et al., 2011). Finally, drugs that inhibit the breakdown of ACh, acetylcholinesterase inhibitors (AChEI), were demonstrated to have beneficial effects on AD patients, with improvements in memory and attention (Brousseau et al., 2007; Pinto et al., 2011).

## Shining new light on the cholinergic system

As discussed above there are important limitations that are inherent to the approach that was taken by most studies. Concerning electrophysiological experiments, it is well known that the spatial and temporal parameters of ACh application are crucial in determining the electrophysiological effects. Given our lack of knowledge about the transmission modes and concentrations of ACh surrounding the receptors, it is very hard to estimate what the effects of ACh on neuronal activity are. In order to advance our knowledge about the way ACh modulates processing in the mPFC it will be crucial to manipulate ACh release from cholinergic terminals, because this is the only way in which we can monitor the postsynaptic effects that occur with realistic cholinergic stimulation. When it comes to the role of ACh in behavior, there are also certain limitations with the pharmacological and knock-out approach. Pharmacology suffers from a lack of specificity, as it stimulates receptors throughout the body and also here the temporal aspects of receptor activation are far from what is physiologically relevant. As mentioned before, animals lacking specific receptors often show compensatory and developmental effects and therefore do not allow us to study the role of receptors in the normal situation.

Fortunately, there are new methods that will allow us to press forward our understanding of the cholinergic modulation of the mPFC by manipulating ACh release from cholinergic neurons themselves and by measuring the release of ACh and the activity of the cholinergic innervation. Two methods that will be crucial are optogenetics (Zhang et al., 2007; Fenno et al., 2011; Yizhar et al., 2011) and the measurement of presynaptic activity with new calcium dyes (Chen et al., 2013; Kaifosh et al., 2013).

Optogenetics makes use of genetically encoded opsins that allow experimenters to stimulate or inhibit the activity of specific populations of neurons. The neurons that are effected can be defined by their genetic background, their location, their projection targets or a combination of these (Josh Huang and Zeng, 2013). Using this method it will be possible to determine the effect of ACh release in specific brain structures. Since release can be both inhibited and stimulated at specific time points during behavioral tests, it will be possible to determine the effects of different release modes in specific brain regions. In addition, electrophysiological effects of ACh release can be measured using *in vitro* or *in vivo* preparations. The power of this approach has already been demonstrated in a number of studies that investigated polysynaptic effects of ACh release (Arroyo et al., 2012; Bennett et al., 2012).

In addition, very sensitive calcium dyes have been developed (Chen et al., 2013) that make it possible to measure presynaptic activity. In other words, if these dyes are expressed in cholinergic neurons of the basal forebrain, it will be possible to measure the activity of their axons in the cortex. This will most likely lead to breakthroughs in our knowledge about the activity of these neurons, as at the moment very little is known about the activity of these fibers. Recently, a similar approach was used on the GABAergic projections from the basal forebrain to the hippocampus, thereby showing for the first time when these axons are active during behavior (Kaifosh et al., 2013).

These methods will make it possible to address key questions in the field of the cholinergic modulation of the cortex. First of all, they will make it possible to investigate when ACh is released and through what kind of signaling mode this occurs. In other words, we will be able to find out what the role is of tonic and phasic release of ACh. In addition, the spatial specificity of cholinergic signaling can finally be addressed. At the moment there is a scarcity of information regarding the degree of specificity of ACh release. For example, currently it is unknown whether ACh release occurs simultaneously throughout the PFC or whether it can be restricted to specific prefrontal areas such as the prelimbic cortex. Moving from a general notion of a role of ACh in attention towards an understanding of when and where exactly ACh is released will be a crucial step towards understanding the cholinergic system.

Since there are multiple sources of ACh, this approach will make it possible to study the role of the basal forebrain, midbrain cholinergic areas and cortical cholinergic interneurons separately. Moreover, cholinergic neurons only make up a small percentage of cortical projections from the basal forebrain (Gritti et al., 1997; Zaborszky et al., 1999; Gritti et al., 2003), and the genetic approach will allow studying the role of these other projections to the cortex, in an approach similar to (Kaifosh et al., 2013). Using optogenetics and genetically encoded calcium indicators will allow researchers to disentangle the role of different cholinergic and basal forebrain neuronal populations.

Also in the field of neurophysiology big advances are to be expected with the development of optical methods. Many of the questions that remained after experiments in acute brain slices can now finally be addressed. In order to understand how ACh modulates processing in the mPFC we will need to deliver ACh in a realistic manner. If we can make cholinergic axons release ACh themselves then we will make a huge step forwards in this respect. As mentioned before, several papers have been published in which this was done (Arroyo et al., 2012; Bennett et al., 2012). It will be necessary to investigate how nicotine affects currents through nAChRs when ACh is not applied with in the bath or with a puff pipette but instead released from cholinergic axons.

Finally, the combination of calcium indicators, allowing us to measure presynaptic activity, and in vivo electrophysiology make it possible to correlate neuronal spiking and field potential dynamics to ACh release. Again, this is expected to provide exciting new insights into the role of ACh in cognition and the cortical mechanisms underlying this.

## Conflict of interest statement

The authors declare that the research was conducted in the absence of any commercial or financial relationships that could be construed as a potential conflict of interest.
